# p38*α* MAPK antagonizing JNK to control the hepatic fat accumulation in pediatric patients onset intestinal failure

**DOI:** 10.1038/cddis.2017.523

**Published:** 2017-10-12

**Authors:** Yongtao Xiao, Jun Wang, Weihui Yan, Kejun Zhou, Yi Cao, Wei Cai

**Affiliations:** 1 Department of Pediatric Surgery, Xin Hua Hospital, School of Medicine, Shanghai Jiao Tong University, Shanghai, China; 2 Shanghai Institute of Pediatric Research, Shanghai, China; 3 Shanghai Key Laboratory of Pediatric Gastroenterology and Nutrition, Shanghai, China

## Abstract

The p38*α* mitogen-activated protein kinase (MAPK) has been related to gluconeogenesis and lipid metabolism. However, the roles and related mechanisms of p38*α* MAPK in intestinal failure (IF)-associated liver steatosis remained poor understood. Here, our experimental evidence suggested that p38*α* MAPK significantly suppressed the fat accumulation in livers of IF patients mainly through two mechanisms. On the one hand, p38*α* MAPK increased hepatic bile acid (BA) synthesis by upregulating the expression of the rate-limiting enzyme cholesterol 7-*α*-hydroxylase (CYP7A1) and peroxisome proliferator-activated receptor *γ* coactivator-1*α* (PGC-1*α*), which in turn activated the transcription of the CYP7A1. On the other hand, p38*α* MAPK promoted fatty acid (FA) *β*-oxidation via upregulating peroxisome proliferator-activated receptor alpha (PPAR*α*) and its transcriptional target genes carnitine palmitoyltransferase 1A (CPT1A) and peroxisomal acyl-coenzyme aoxidase 1 (ACOX1). Dual luciferase assays indicated that p38*α* MAPK increased the transcription of PPAR*α*, PGC-1*α* and CYP7A1 by upregulating their promoters’ activities. In addition, *in vitro and in vivo* assays indicated p38*α* MAPK negatively regulates the hepatic steatosis by controlling JNK activation. In conculsion, our findings demonstrate that hepatic p38*α* MAPK functions as a negative regulator of liver steatosis in maintaining BA synthesis and FAO by antagonizing the c-Jun N-terminal kinase (JNK).

Children with intestinal failure (IF) most caused by intestinal dysmotility disorders or short bowel syndrome, and require long-term parenteral nutrition (PN) for survival. The bowel dysfunction and long-term PN often results of IF-associated liver disease (IFALD), which is a major complication and the leading cause of morbidity and mortality in pediatric IF patients.^1, 2, 3^ IFALD is characterized initially by intrahepatic cholestasis, and then by progressive portal inflammation to steatosis and fibrosis during PN. Liver steatosis and fibrosis persist in a majority of patients even after weaning off PN. Although multiple risk factors including limited amount of enteral nutrients, duration and composition of PN, different components of PN, prematurity, low birth weight, bacterial overgrowth, and massive intestinal resection link to the IFALD,^3, 4, 5^ the mechanisms causing and maintaining hepatic steatosis in IF patients are largely unclear.

The p38 mitogen-activated protein kinases (MAPKs) are important regulators of cellular responses to a variety of extracellular stimuli. The p38 MAPK family includes four members (p38*α*, p38*β*, p38*γ* and p38*δ*),^6^ in which p38*α* is the predominant isoform in liver.^7^ It has been reported that mice with liver-specific deletion of p38*α* exhibited enhanced hepatocyte proliferation after partial hepatectomy.^8, 9^ The hepatic p38*α* has shown to repress cell proliferation by antagonizing the c-Jun N-terminal kinase (JNK)/c-Jun pathway.^9, 10^ In addition, p38*α* has been shown to inhibit JNK activation to prevent endotoxin-induced liver failure.^11^ Activation of p38 has been observed in the livers of mouse models of obesity, and hyperlipidemia^12, 13^ It has been demonstrated that p38 might have a regulatory role in hepatic gluconeogenesis and lipogenesis.^14, 15, 16, 17^ We here showed that p38*α* MAPK was activated in livers of IF patients and related to the development of steatosis. We thus hypothesized that p38*α* MAPK may have an important role in the causing or maintaining steatosis in IF patients.

The bile acid (BA) synthesis and fatty acid (FA) fatty acid *β*-oxidation (FAO) in the liver are two predominant pathways for cholesterol and lipid homeostasis. Cholesterol 7-*α*-hydroxylase (CYP7A1) is the rate-limiting enzyme that commits cholesterol to the neutral bile acid biosynthesis pathway and is highly regulated. It has been reported previously that mice overexpressing Cyp7a1 is protected against high fat diet-induced hypercholesterolemia, obesity and insulin resistance.^18, 19^ It has been found that peroxisome proliferator-activated receptor *α* coactivator-1*α* (PGC-1*α*) is activated by p38*α* MAPK^20^ and that PGC-1*α* activates CYP7A1 expression in activation of the CYP7A1 promoter.^21^ Thus, p38*α* MAPK might activate CYP7A1 expression in activation of the CYP7A1 promoter in part through PGC-1*α*. The nuclear receptor peroxisome proliferator-activated receptor *α* (PPAR*α*) has been shown to serve as a key transcriptional regulator of the FAO pathway. As transcription factors, PPAR*α* has critical roles in hepatic FAO mainly through regulating canonical target genes carnitine palmitoyltransferase 1A (CPT1A) and peroxisomal acyl-coenzyme aoxidase 1 (ACOX1).^22, 23^Recently, evidence has emerged that the p38*α* MAPK could phosphorylated and activated the transcription factor PPAR*α*, leading to enhanced ligand-mediated coactivation by the transcriptional coactivator PGC-1*α* in cardiac myocytes.^24^ Therefore, p38 MAPK may be also involved in FAO by regulating the PPAR*α* and PGC-1*α*.

Given that the p38 MAPK has important roles in FAO and BA synthesis, we hypothesized that p38*α* MAPK may be a critical regulator in IF-associated liver steatosis. In present study, we systematically explored the role of p38*α* MAPK in the development of IF-associated hepatic steatosis and identified the involved targets and pathways, indicating that hepatic p38*α* MAPK represents an exciting pharmacological target for the treatment of IFALD

## Results

### The fat accumulation in livers of pediatric IF patients was associated with PN duration

**Figure 1:**
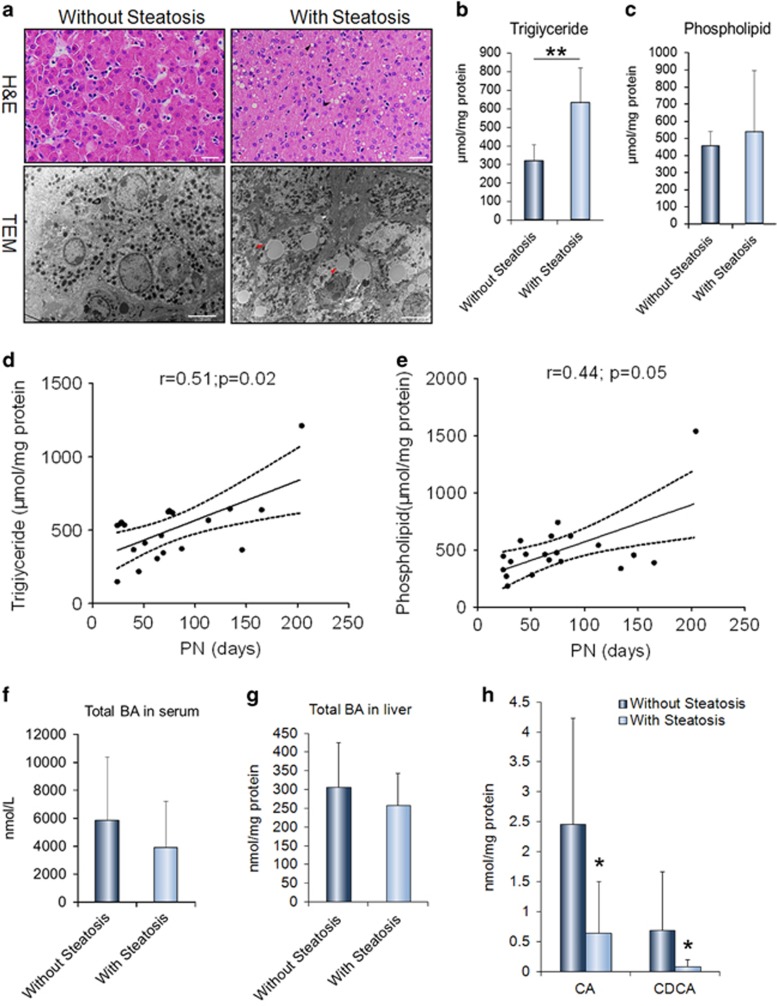
Liver steatosis correlated with the duration of parenteral nutrition (PN) and related to the reducing bile acid (BA) contents in the liver tissues of in pediatric intestinal failure (IF) patients. (**a**) Representative images of haematoxylin and eosin (H&E) and transmission electron microscopy (TEM) in the liver tissues of IF patients with or without steatosis. (**b**,**c**) Quantification of the levels of hepatic triglyceride (TG) and phospholipids (PL) in IF patients. (**d**,**e**) The contents of hepatic TG and PL were positively correlated with PN duration. (**f**,**g**) The levels of BA in the liver and serum reduced in the patients with steatosis compared with the ones without steatosis. (**h**) The primary BA cholic acid (CA) and chenodeoxycholic acid (CDCA) decreased in the livers of patients with steatosis, related to the ones without steatosis. Scale bar=50 μm, 2 μm; Arrows indicate the fatty drops (**a**) with steatosis (*n*=6–10) and without steatosis (*n*=8–14) *P<0.05, **P<0.01

A total of 24 patients at median age 4.0 months (IQR 2.25–6) were enrolled in this study (Table 1). Causes of IF included small bowel atresia (*n*=6), necrotizing enterocolitis (*n*=7), mid-gut volvulus (*n*=3), chronic intestinal pseudo-obstruction (*n*=5) and aganglionosis of hirschsprung’s disease (*n*=3). Overall, 42% (10/24) of patients had liver steatosis (Table 1). In patients with steatosis, about equal amounts of micro- and macrovesicular steatosis were found in the liver sections with histological examination (Figure 1a). In line with histological changes, the patients with steatosis had significantly increased levels of hepatic triglyceride (TG) and phospholipids (PL), compared with those without steatosis (Figures 1b and c). As expected, the serum lipids including LDL cholesterol and TG elevated significantly in patients with steatosis, related to that without steatosis (Table 1). In addition, the number of apoptotic hepatocytes and degrees of portal fibrosis were higher in liver sections from patients with steatosis when compared with those without steatosis (Supplementary Figure 1). As showed in Table 1, the patients with steatosis had been longer on PN as compared with those without steatosis (IQR 40 (27–75) *versus* 100 (56.75–143), *P*=0.026, Table 1). Correlated analysis showed that hepatic contents of TG (*r*=0.51, *P*=0.02) and PL (*r*=0.44, *P*=0.05) were positively correlated with PN duration, indicating the PN is the major cause of liver steatosis in IF patients (Figures 1d and e). It is known that hepatic bile acid (BA) synthesis is an important pathway for cholesterol catabolism in the liver. We here showed that the total BA in serum and liver, as well as plasma bilirubin, decreased evidently in IF patients with steatosis compared with those without steatosis (Figures 1f and g; Table 1). The BA composition of liver and serum was markedly altered in patients with steatosis, exhibiting a significant reduction in the proportion of the primary BA, such as cholic acid (CA) and chenodeoxycholic acid (CDCA) (Figure 1h; Supplementary Table 4).

### Downregulation of p38*α* MAPK and upregulation of JNK in steatotic livers of pediatric IF patients

**Figure 2:**
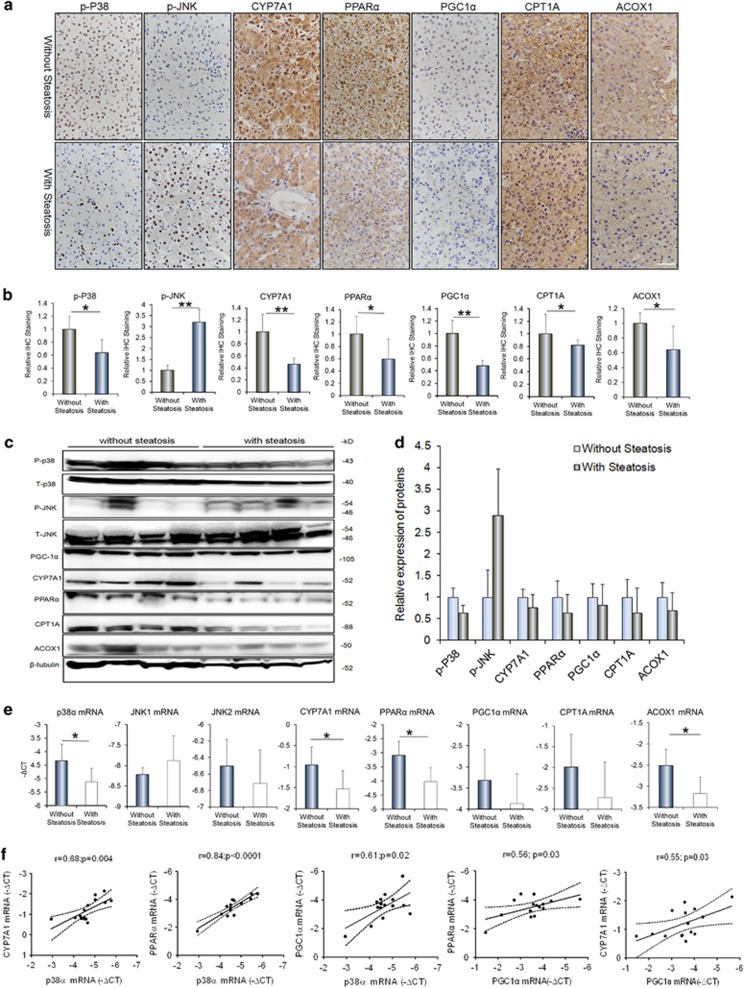
The p38*α* MAPK activation was decreased in livers of IF patients with steatosis and associated with expression of cholesterol 7-*α*-hydroxylase (CYP7A1), proliferator-activated receptor *α* coactivator-1 (PGC-1*α*) and nuclear receptor peroxisome proliferator-activated receptor *α* (PPAR*α*). (**a**) Representative images of p-p38, p-JNK, CYP7A1, PGC-1*α*, PPAR*α*, CPT1A and ACOX1 stainings in the liver tissues of IF patients without steatosis (*n*=14) and with steatosis (*n*=10). (**b**) Quantification of the results in panel A. (**c**) Western blot analyses of the p-p38, p-JNK, CYP7A1, PGC-1*α*, PPAR*α*, CPT1A and ACOX1 protein levels in the livers of IF patients with or without steatosis. (**d**) Quantification of the protein results in panel C. (**e**) qRT- PCR analyses determine the levels of p38*α*, JNK1/2, CYP7A1, PGC-1*α*, PPAR*α*, CPT1A and ACOX1 mRNAs in the IF patients. (**f**) The relationships between p38*α* mRNA and mRNA levels of CYP7A1, PGC-1*α*, PPAR*α*, and between PGC-1*α* mRNA and CYP7A1 mRNA, PPAR*α* mRNA in the liver tissues of the IF patients with Pearson’s correlations. Scale bar=25 μm *P<0.05, **P<0.01

To investigate the potential roles of p38*α* MAPK in hepatic steatosis, the expression and activation of p38*α* MAPK were examined firstly in liver samples from pediatric IF patients. As shown in Figure 2, the levels of phosphorylated p38*α* MAPK (Thr180/Tyr182) were decreased significantly in liver sections from patients with steatosis, relative to ones without steatosis (Figures 2a and b). In contrast, we here showed that the phosphorylated levels of JNK (Thr183/Tyr185) were increased evidently in liver samples from patients with steatosis compared with those without steatosis (Figures 2a and b). Western blot analysis on liver samples further confirmed the significant reduction of phosphorylated p38*α* MAPK and elevation of phosphorylated JNK in the livers of patients with steatosis, relative to ones without steatosis (Figures 2c and d). Consistent with the changes in protein levels, the expression of p38*α* MAPK mRNA was decreased and JNK mRNA was increased in the livers of patients with steatosis, compared with the ones without steatosis (Figure 2e).

### Hepatic p38*α* MAPK was related to BA synthesis and FAO in livers of pediatric IF patients

Considering that the hepatic BA synthesis and FAO are vital pathways for fatty acid catabolism in the liver, we determined the expression of the key genes involved in these pathways. Cholesterol 7-*α*-hydroxylase (CYP7A1) is the key enzyme for the conversion of cholesterol to bile acids. The peroxisome proliferator-activated receptor alpha (PPAR*α*) and its transcriptional target genes carnitine palmitoyltransferase 1 A (CPT1A) and peroxisomal acyl-coenzyme aoxidase 1 (ACOX1) have critical roles in hepatic FAO. The peroxisome proliferator activator receptor *γ* coactivator-1 alpha (PGC-1*α*) is an important coactivator in the fatty acid/bile acid metabolism. As shown in Figure 2, both IHC analysis and western blot assays showed the proteins of CYP7A1, PGC-1*α*, PPAR*α*, CPT1A and ACOX1 were decreased in livers tissues from patients with steatosis when compared with ones without steatosis (Figures 2a–d). In line with these proteins changes, the quantitative real-time PCR (qRT-PCR) also showed these genes mRNA reduced in steatotic livers (Figure 2e). In addition, we found a significant positive correlation between the mRNA levels of p38*α* and CYP7A1 (*r*=0.68, *P*=0.004), of p38*α* and PPAR*α* (*r*=0.84, *P* <0.0001) and of p38*α* and PGC-1*α* (*r*=0.61, *P*=0.02) in all liver samples from IF patients (Figure 2f). Correlated analysis also showed that mRNA levels of PGC-1*α* were positively correlated with levels of PPAR*α* mRNA (*r*=0.56, *P*=0.03) and CYP7A1 mRNA (*r*=0.55, *P*=0.03) (Figure 2f).

### p38*α* MAPK and JNK antagonistically regulates the PN-induced liver steatosis

**Figure 3:**
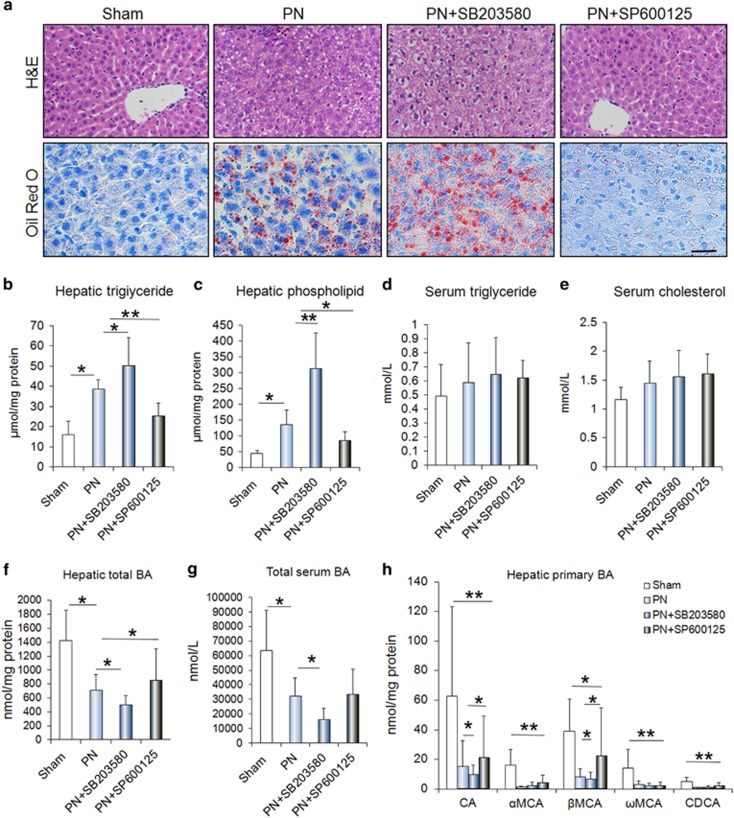
p38*α* MAPK and JNK have opposite effects on PN-associated hepatic steatosis. (**a**) H&E and Oil Red O staining showed that p38*α* MAPK inhibition with SB203580 treatment increased the fat accumulated in the liver of PN-fed rats. In contrast, JNK inhibition with SP600125 treatment suppressed the PN-associated steatosis. (**b–e**) The alteration of levels of the triglyceride (TG) and phospholipids (PL) in liver and serum from groups of Sham, PN, PN+SB203580 and PN+SP600125. (**f**,**g**) The levels of total BA in the liver and serum from groups of Sham, PN, PN+SB203580 and PN+SP600125. (**h**) The measurement of primary BA including cholic acid (CA), chenodeoxycholic acid (CDCA) and *α*,*β*,*ω*-muricholic acid (*α*-,*β*-,*ω*MCA) in livers from groups of Sham, PN, PN+SB203580 and PN+SP600125. Scale bar=25 μm *P<0.05, **P<0.01

PN is a lifesaving therapy in patients with intestinal failure. However, others and we both indicated that the PN is the main contributor of liver steatosis in IF patients. Thus, we are to determine the relevance of p38*α* MAPK and JNK in hepatic steatosis in IF patients based on the model of PN. As shown in Figure 3a, the hematoxylin & eosin (H&E) and Oil Red O-stained analysis showed that the fat in the form of micro- and macrovesicular steatosis accumulated in liver after six days of continuous PN administration (Figure 3a). In consistent with the histological appearance of steatosis, PN significantly induced hepatic TG and PL (Figures 3b and c). Interestingly, serum TG and cholesterol contents were not affected by PN administration (Figures 3d and e; Supplementary Table 6). With measurement of the BA, we found that found that the total BA in the liver and serum were decreased significantly in PN-fed rats compared with the controls (Figures 3f and g; Supplementary Table 7). The primary BA including cholic acid (CA), chenodeoxycholic acid (CDCA) and *α*, *β*, *ω*-muricholic acid (*α*-, *β*-, *ω*MCA) in livers of PN-rats reduced markedly, relative to the controls (Figure 3h; Supplementary Table 7). To evaluate the role of hepatocyte p38*α* MAPK and JNK in PN-induced steatosis, we treated the PN-rats with SB203580 and SP600125, which are specific inhibitors for p38*α* MAPK and JNK. As indicated in Figure 3, the livers of SB203580-treated rats exhibited significantly increased fatty and lipid accumulation, whereas the livers of SP600125-treated rats presented the opposite trend compared with their corresponding PN-fed rats, as analyzed by H&E, Oil Red O-stained liver sections and direct measurement of hepatic TG and PL. Furthermore, SB203580 treatment exacerbated PN-decreased BA synthesis in the liver, whereas the SP600125 treatment promoted the hepatic BA production (Figures 3f–h; Supplementary Table 7).

**Figure 4:**
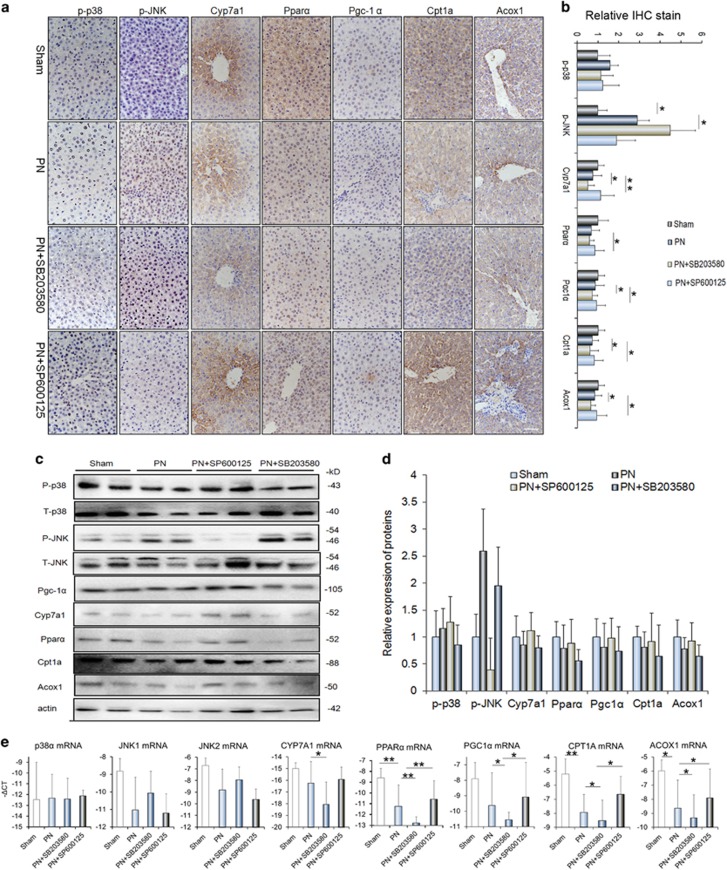
p38*α* MAPK opposing JNK to suppress the expression of CYP7A1, PGC-1*α*, PPAR*α*, CPT1A and ACOX1 *in vivo*. (**a**) Representative images of p-p38, p-JNK, CYP7A1, PGC-1*α*, PPAR*α*, CPT1A and ACOX1 stainings in the liver tissues of Sham, PN, PN+SB203580 and PN+SP600125 groups. (**b**) Quantification of the results in panel A. (**c**) Western blot analyses of the p-p38, p-JNK,CYP7A1, PGC-1*α*, PPAR*α*, CPT1A and ACOX1 protein levels in the livers of Sham, PN, PN+SB203580 and PN+SP600125 groups. (**d**) Quantification of the protein results in panel C. (**e**) qRT-PCR analyses determine the levels of p38*α*, JNK1/2, CYP7A1, PGC-1*α*, PPAR*α*, CPT1A and ACOX1 mRNAs in the livers of Sham, PN, PN+SB203580 and PN+SP600125 groups. Scale bar=25 μm *P<0.05, **P<0.01

We next sought to explore the potential pathways underlying the effects of p38*α* MAPK and JNK on PN-mediated hepatic steatosis. Due to BA synthesis and FAO functions as important pathways in fatty acid metabolism, we therefore investigated the effects of involvement of p38*α* MAPK and JNK on key genes that regulating BA synthesis and FAO during PN-mediated hepatic steatosis. As shown in Figure 4, SB203580 and SP600125 treatment abrogated the PN-induced activation of p38*α* MAPK and JNK via reducing the phosphorylated levels of them, as indicated by IHC and western blots analysis (Figures 4a–d). Moreover, we noticed that SB203580 treatment increased activation of JNK, wherever, JNK inhibition did not affect the p38*α* MAPK, suggesting p38*α* MAPK may suppress the activation of JNK (Figures 4a–d). IHC analysis and Western blot assay showed the proteins of CYP7A1, PGC-1*α*, PPAR*α*, CPT1A and ACOX1 in the liver reduced evidently following the SB203580 treatment, on the contrary, SP600125 treatment had opposite effects on these proteins expression (Figures 4a–d). Consistent with the alteration in protein levels, the expression of CYP7A1 mRNA, PGC-1*α* mRNA, PPAR*α* mRNA, CPT1A mRNA and ACOX1 mRNA was decreased after p38*α* MAPK and was increased in the presence of JNK inhibition (Figure 4e).

### p38*α* MAPK and JNK antagonistically controls free fatty acid-induced cellular steatosis

**Figure 5:**
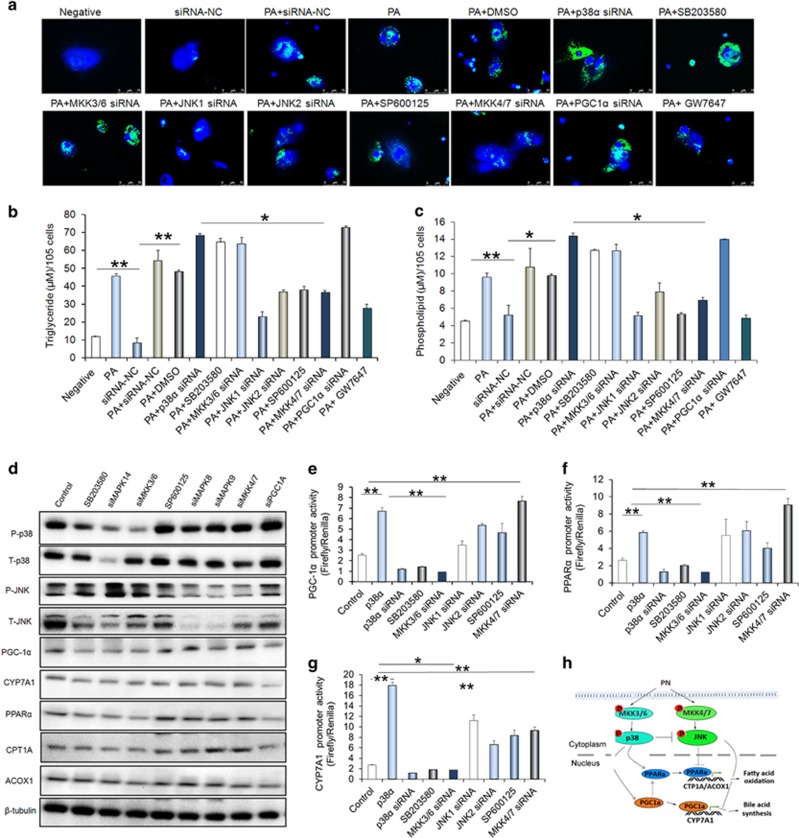
p38*α* MAPK antagonistically JNK to control the palmitate (PA)-mediated lipogenesis. (**a**) Representative images of neutral lipids staining in the indicated cells. (**b**,**c**) Quantification of the contents of hepatic triglyceride (TG) and phospholipids (PL) in indicated treatments. (**d**) Western blot analyses for the p-p38, p-JNK, CYP7A1, PGC-1*α*, PPAR*α*, CPT1A and ACOX1 protein levels in the human primary hepatic cell with indicated treatments. (**e–g**) Relative promoter activity of CYP7A1, PGC-1*α* and PPAR*α* with indicated treatments. (**h**) The scheme illustrates a potential mechanism by which p38*α* MAPK controls the fatty acid metabolism in the livers of IF patients. Scale bar=25 μm *P<0.05, **P<0.01

As the saturated fatty acids are the significant components of parenteral formulations, we used PA to induced cellular steatosis and further to investigated roles of p38*α* MAPK in cellular steatosis. As shown in Figure 5, the images showed that the PA-induced lipid and fat droplets in the human primary liver cells (Figure 5a), which was consistent with a previous study.^25^ Moreover, the PA treatment significantly increased levels of intracellular TG and PL (Figure 5b). We next to determine whether p38*α* MAPK are responsible for the steatosis that observed in PA treated human primary hepatocytes. We showed that SB203580 treatment or MAPK14 knockdown effectively reduced the phosphorylation of p38*α* MAPK and promoted the PA-mediated hepatic steatosis, as analyzed by neutral lipid stained liver cells and direct measurement of hepatic TG and PL (Figures 5a–d). We next to showed that JNK inhibitor SP600125 or JNK knockdown prevented PA-induced hepatic steatosis by reducing the contents of TG and PL and neutral lipid stains (Figures 5a–d). Because the p38*α* MAPK are activated by MKK3 and MKK6 and JNK are directly activated by MKK4 and MKK7, we thus knockdown the MKK3/6 and MKK4/7 respectively to investigate possible mechanisms of p38*α* MAPK and JNK involved in the liver steatosis. Indeed, MKK3/6 knockdown and MKK4/7 knockdown abrogated the activation of p38*α* MAPK and JNK. In addition, MKK3/6 knockdown increased the PA-mediated steatosis, while MKK4/7 knockdown decreased the PA-associated steatosis (Figures 5a–d). We further showed that the PPAR*α* agonist GW7647 treatment significantly attenuated the PA-mediated steatosis and. increased the expression of CPT1A and ACOX1 (Figures 5a–d). PGC-1*α* has an important role in co-ordinating transcriptional programmes of FAO.^26, 27^ We here showed that PGC-1*α* knockdown increased the PA-induced steatosis, as indicated by neutral lipid stains and TG and PL measurement. Moreover, the western blot analysis showed that PGC-1*α* knockdown reduced the expression of PPAR*α* as well as its targets ACOX1, CPT1A and bile acid synthesized enzyme CYP7A1 (Figure 5d).

### p38*α* MAPK antagonizing JNK to promote the transcription of PGC-1*α*, CYP7A1 and PPAR*α*

As shown in Figure 5d, we firstly noticed that p38*α* MAPK inhibition significantly increased the JNK activation, indicating p38*α* MAPK has inhibitory effects on the JNK activation in the hepatocytes. Secondly, we indicated that p38*α* MAPK activation increased expression of PGC-1*α*, PPAR*α* and CYP7A1, while the JNK. activation has opposite effects on these proteins expression (Figure 5d). To understand the molecular mechanisms involved, we constructed luciferase vectors contained promoters of PGC-1*α*, PPAR*α* and CYP7A1 and determined activities of these promoters response to the p38*α* MAPK and JNK activation. As shown Figure 5, the p38*α* overexpression enhanced significantly the luciferase activities of PGC-1*α*, PPAR*α* and CYP7A1 promoters, while p38 MAPK inhibition with MAPK14, MKK3/6 knockdown or SB203580 treatment abolished the activities of these gene promoters (Figures 5e–g). For JNK transcriptional regulating PGC-1*α*, PPAR*α* and CYP7A1, JNK inhibition with JNK, MKK4/7 knockdown or SP600125 treatments significantly increased the activities of promoters of them, indicating JNK activation may have suppressive role in transcription of PGC-1*α*, PPAR*α* and CYP7A1 (Figures 5e–g).

## Discussion

In this study, we firstly indicated that pediatric IF patients exhibited liver steatosis and was coupled with degrees of fibrosis and hepatic apoptosis. The liver steatosis in patients was tightly associated with the PN duration. Secondly, the downregulation of fatty acid *β*-oxidation (FAO) and bile acid (BA) contributed to the liver steatosis in pediatric IF patients. Thirdly, p38*α* MAPK and JNK antagonistically controls FAO and BA synthesis to affect the hepatic fat accumulation in pediatric IF patients by regulating the CYP7A1 and PPAR*α*.

In a previous study by Annika and co-workers,^28^ prevalence of hepatic steatosis during PN was 60% in pediatric onset IF. We here showed that about 40% pediatric IF (10/24) patients had liver steatosis with much lower ages. It reported that the younger patients with PN are more prone to cholestasis. It thus suggests the ages of patients maybe account for differences of prevalence in hepatic steatosis. Similar to the previous findings, both micro- and macrovesicular steatosis was observed in liver of patients with steatosis. The liver steatosis was associated with progression of portal fibrosis and hepatic apoptosis in patients with IF. It also showed that patients with liver steatosis had been longer on PN as compared with those without steatosis, which suggests long-term PN is the key factor accounting for the liver steatosis in IF patients. In addition, we found that the IF patients with steatosis exhibited decreased levels of primary BA in both blood and liver, including CA and CDCA, concurrent with decreased levels of secondary BA. In human, the primary BA, including CA and CDCA, were converted from the cholesterol mainly via the cholesterol 7-*α* hydroxylase (CYP7A1). CYP7A1 is the rate-limiting enzyme in the bile acid biosynthetic pathway in the liver and thus controls cholesterol and bile acid homeostasis. It has been reported that overexpression of CYP7A1 in mouse liver prevents lithogenic diet-induced atherosclerosis and maintains cholesterol homeostasis.^18, 19^ We here indicated that expression of CYP7A1 protein as well as mRNA was decreased in steatotic livers as compared with ones without steatosis. It means that BA synthesis decreasing may cause the fat accumulated in the patients with steatosis. Except the BA synthesis, fatty acid *β*-oxidation (FAO) has important role in fatty acid metabolism via degradation of activated FA to acetyl-CoA. PPAR*α* is a ligand-activated nuclear receptor highly expressed in the liver, which controls gene expression levels of the rate-limiting enzymes of peroxisomal *β*-oxidation, including ACOX1 and CPT1A. In this study, the patients with steatosis had lower levels of PPAR*α*, ACOX1 and CPT1A than the ones without steatosis. We therefore speculated that the decreasing the FAO has a stimulatory role in liver steatosis. PGC-1 has already been shown to be a critical activator of FAO and BA metabolism. In present study, we showed that the expression of PGC-1*α* was downregulated in the livers of patients with steatosis when compared with the ones without steatosis. In addition, the levels of the PGC-1*α* mRNA were positively correlated the levels of PPAR*α* mRNA and CYP7A1 mRNA. It thus suggests that the PGC-1*α* may regulate the FAO and BA synthesis via promoting the expression of PPAR*α* and CYP7A1. Recent studies indicated that the p38*α* MAPK could promote activation and expression of PGC-1*α*^20, 29^ and blockade of p38 MAPK lead to hypertriglyceridemia and fatty liver.^30^ We here showed that the activation of p38 MAPK reduced in steatotic livers and its mRNA levels were tightly correlated to the levels of PGC-1*α* mRNA, PPAR*α* mRNA and CYP7A1 mRNA. We thus postulated that p38 might mediate suppression of liver steatosis through regulating the FAO and BA synthesis. In contrast, we observed that the JNK activation was increased in steatotic livers, indicating it might has opposite roles against p38 MAPK*α* in liver steatosis.

Parenteral nutrition (PN) is a routine clinical procedure to treat pediatric IF patients. Others and we showed that the continuous infusion of PN could cause liver steatosis in IF patients. In this study, we thus established the rat model of PN to explore the possible mechanisms involved in steatosis. The rats with PN for 7 days exhibited steatosis and increasing levels of TG and PL in the liver. How PN infusion did increased levels of hepatic TG and PL? A previous study that PN infusion was not accompanied by increased de novo lipogenesis. In turn, the levels of lipogenic enzymes were decreased in PN-fed rats.^31^ Hence, we postulated PN-induced steatosis more likely results from reduced FAO and BA synthesis. Indeed, the key regulators of FAO including PPAR*α*, CPT1A and ACOX1 reduced in PN-fed rats. Decreased Cyp7a1 expression along with the hepatic primary BA was also observed in PN-fed rats. In order to determine the roles of p38*α* MAPK and JNK in the PN-associated steatosis, we used the SB203580 and SP600125 to inhibit the activation of p38 MAPK and JNK firstly. It was observed that SB203580 treatment aggregated steatosis in PN-fed rats exhibiting increased the levels of TG and PL in liver tissues. Moreover, rats with SB203580 treatment showed a further decreased in BA synthesis and expression of PPAR*α*, CPT1A, ACOX1 and CYP7A1. Unexpectedly, it was observed that SB203580 treatment increased the JNK activation. JNK inhibition with SP600125 suppressed liver PN-associated steatosis. JNK pathway has been shown to have a inhibitory role in regulating PPAR*α* and CYP7A1 levels in primary rat hepatocytes.^32^ Therefore, the p38*α* MAPK and JNK may have opposite effects on steatosis in terms of regulating the FAO and BA synthesis.

Since lipid emulsion of PN rich in medium-chain saturated FA, we further used the PA to induce hepatic lipogenesis *in vitro*. It indicated that PA treatment could increase the number of the lipid droplets and the content of the TG and PL in the liver cells, and these effects were significantly aggravated by the p38 MAPK inhibition with SB203580 treatment, or MAPK14, MKK3/6 knockdown. Similar to the findings *in vivo*, p38 MAPK inhibition suppressed the expression of PPAR*α*, PGC-1*α* and CYP7A1. Recently, several studies showed that p38*α* MAPK could inhibit JNK activation in liver.^10, 11, 33^ Consistent with these studies, we showed that the JNK activation was indeed increased after p38*α* MAPK inhibition. We further found that JNK inhibition could abolish the PA-mediated liver lipogenesis. To further define the mechanism of p38*α* MAPK in hepatic lipogenesis, we examined the role of p38*α* MAPK in activation of the PGC-1*α*, PPAR*α* and CYP7A1 promoters. We here showed that the promoters of PGC-1*α*, PPAR*α* and CYP7A1 were stimulated by p38*α* overexpression as expected, but they were suppressed by the inhibition of p38 via SB203580 treatment or MAPK14, MKK3/6 knockdown. Because p38*α* MAPK could phosphorylate PGC-1*α* and PPAR*α* sites,^20, 24^ we hypothesized that p38*α* MAPK enhanced their transcription maybe via phosphorylated them. It has been shown that PGC-1*α* is activated by p38 kinase^20^ and that PGC-1*α* activates CYP7A1 expression.^21^ Thus, p38 MAPK might activate CYP7A1 expression through PGC-1*α*. In contrast to effects of p38 MAPK on PGC-1*α*, PPAR*α* and CYP7A1 promoters, JNK activation significantly suppressed the activities of these promoters, which was consistent with the previous reported.

In summary, the present studies demonstrate that p38*α* MAPK has a inhibitory role in liver steatosis of IF patients via increased the FAO and BA synthesis by antagonizing JNK. We further identify PGC-1*α* as a target of the p38*α* MAPK and a requirement for PPAR*α* and CYP7A1 expression.

## Materials and methods

### Patients

A total of 24 pediatric patients with IF were enrolled in this study. The serum samples and liver specimens were obtained from patients who underwent surgery. All patients’ guardians provided written informed consent. The patients’ characteristics are presented in Table 1. This study was approved by the Faculty of Medicine’s Ethics Committee of Xin Hua hospital (XHEC-C-2016-063), School of Medicine, Shanghai Jiao Tong University, Shanghai, China. All methods in this study were carried out in accordance with the relevant guidelines.

### Model of PN

Three-week old male Sprague-Dawley rats (Animal Experiment Center of the Chinese Academy of Science) were housed in individual cages and exposed to a 12-h light–dark cycle for seven days. Rats were randomized to sham group (*n*=10), PN group (*n*=8), PN plus SB203580 group (PN+SB203580, *n*=8) and PN plus SP600125 group (PN+ SP600125, *n*=6). The catheters for PN were placed into the rats’ external jugular veins and infused with 30 ml/day PN solution for six days. Sham group received exactly same process and infused with saline and fed with standard chow *ad libitum*. The groups of PN+SB203580 and PN+SP600125 were administrated with SB203580, SP600125 at concentration of 2 mg/kg/day, 5 mg/kg/day via catheters. Composition of TPN solution is presented in Supplementary Table 1 as described previously.^34^ All experiments were approved by the Animal Care Committee of Xin Hua hospital, Shanghai Jiao tong University.

### Analysis of triacylglycerols, phospholipids, neutral lipid and bile acid

The quantitative estimation of triglyceride or phospholipids was determined using EnzyChrom^TM^ Triglyceride Assay Kits (BioAssay Systems, Hayward, CA, USA) and EnzyChrom^TM^ Phospholipid Assay Kits (BioAssay) according to the manufacturer’s protocols. The LipidTOX™ neutral lipid stains (Thermofisher scientific, Molecular Probes, Eugene, OR, USA) was used to monitor the content of neutral lipids in liver cells as described in the manufacturer’s protocols. For analyzing the profiles of bile acids, the detail methods were described in Supplementary Methods.

### Steatosis analyses, fibrosis determination and immunohistochemistry

Steatosis analyses were using hematoxylin and eosin (H&E) staining and Oil Red O staining. Steatosis was determined blindly based on the proportion of Oil hepatocytes affected (0= absent, 1≤25%, 2=25–50%, 3≥50%) as described previously.^28^ Fibrosis was performed using mason’s trichrome stain according to the manufacturer’s protocol (Genmed Scientifics, Wilmington, DE, USA). The liver fibrosis was assessed by Metavir fibrosis stage (0=absent, 1=fibrous expansions of most portal areas, 2=focal portal-to-portal bridging, 3=marked bridging and 4=cirrhosis)^35^ Immunohistochemistry was performed using the diaminobenzidine (DAB) chromogen as described previously.^36^ Briefly, paraffin-embedded tissues were deparaffinized using xylol and descending concentrations of ethanol. The citrate buffer (pH 6.0 or pH 8.0) was used for antigen retrieval. Endogenous peroxidases were removed by 0.3% H_2_O_2_ and then bloced using 10% bovine serum albumin (BSA). Primary antibodies were applied in an optimal concentration overnight in a wet chamber (Phosphorylated p38 (Cell Signaling, Danvers, MA, USA, 4511), dilution 1:100; Phosphorylated JNK (Cell Signaling, 4668), dilution 1:100; CYP7A1 (Millipore, Darmstadt, Germany, MABD42), dilution 1:100; PPAR*α* (Abcam, Bristol, UK, ab8934) dilution 1:100; FXR (Invitrogen,Camarillo, CA, USA, 417200), dilution 1:500; CPT1A (Proteintech, Rosemont, IL, USA, 15184-1-AP), dilution 1:100; ACOX1 (Proteintech, 10957-1-AP), dilution 1:100 PGC-1*α* (Abcam, ab54481), dilution 1:200). Antibody binding was visualized by a liquid DAB Substrate Chromogen System (Dako, Glostrup, Denmark). The IHC images analysis was used software Image Pro Plus (Media Cybernetics) 10 fields/sample.

### Cell culture and treatments

The primary human hepatocytes (F00995-P) were obtained from Research Institute for Liver Disease (Shanghai, China). Transient transfections with small interfering RNAs (siRNAs) were performed using Lipofectamine® RNAiMAX Transfection Reagent (ThermoFisher) following the manufacturer’s protocol. The small interfering RNA (siRNA) duplexes of MAPK14, MAPK8, MAPK9, MKK3/6, MKK4/7 and PPARGC1A were synthesized by GenePharma (Shanghai, China). The sequences of siRNAs were listed in Supplementary Table 2. After 28 h of transfection, the 400 *μ*M palmitate (PA) treated the primary hepatocytes for 18 h. The SB203580 (20 *μ*M), SP600125 (10 *μ*M) or GW7647 (2 *μ*M) treated the liver cells prior 1 h to PA adding and last 16 h.

### Quantitative real-time PCR and western blot

Total RNA was extracted with Trizol according to the protocol of the manufacture (Invitrogen, Foster, CA, USA). A SYBR-Green Universal Master Mix kit and a High Capacity cDNA Reverse Transcription kit were employed to detect the levels of the genes. The primers are listed in Supplementary Table 3. For Western blot, the equal amounts of proteins (200 *μ*g/well) were separated by 4-12% SDS-PAGE, and transferred to nitrocellulose membranes. The membranes were incubated overnight at 4 °C with primary antibodies. Antibodies for GAPDH, CYP7A1, PPAR*α*, p-p38, p-JNK, PGC-1*α*, ACOX1 and CPT1A were analyze. The membranes were washed with PBS (containing 0.1% Tween) and incubated with horseradish-peroxidase conjugated detected the antigen-antibody complexes using an ECL Plus chemiluminescence reagent kit (Pierce, Rockford, IL, USA).

### Reporter analysis

To construct reporter vectors carrying promoters of CYP7A1, PPARA and PPARGC1A, we synthesized the fragments containing the promoters for human CYP7A1, PPARA and PPARGC1A that were showed in Supplementary Methods, and cloned them into the psiCHECK2 luciferase vector (Promega, Madison, WI). The promoter vectors were cotransfected with siRNAs for MAPK14, MAPK8, MAPK9, MKK3/6, MKK4/7 or a negative control into COS-7 cells using Lipofectamine 2000. The cell lysates were harvested at three days after transfection, and the reporter activity was measured with the Dual Luciferase Assay (Promega). The firefly luciferase values were normalized to Renilla, and the firefly/Renilla ratios are presented.

### Statistical analysis

Data statistics are presented as medians IQR or mean±S.D. For comparisons of different groups, statistical significance was determined based on the Student’s *t*-test. Correlations were tested with Spearman rank correlation. *P*-values <0.05 were considered statistically significant.

## Figures and Tables

**Table 1:** Patient characteristics, liver biochemistry, serum lipids and serum bilirubin in intestinal failure patients with and without liver steatosis

	**All patients**	**Patients without steatosis**	**Patients with steatosis**	*P*-**value**
Patients (n)	24	14	10	
Age (months)	4 (2.25–6)	4 (1.01–4)	7.5 (3.5–12)	0.136
Gestation age (weeks)	37 (34–38)	37 (34–38)	36.5 (34.25–38)	0.839
Gestation weight (g)	3000 (2490–3252)	3000 (2580–3050)	3075 (2355–3363.5)	0.982
Left bowel length (cm)	145 (116.75–180)	145 (124.25–180)	117.5 (104.5–146.75)	0.401
PN duration (days)	63 (29.5–104)	40 (27–75)	100 (56.75–143)	0.026
Liver enzymes				
Plasma *γ*-glutamyl-transferase, GGT (U/l)	58 (44.5–140)	58 (38–121)	109.5 (60.75–189.5)	0.205
Plasma alanine aminotransferase, ALT (U/l)	61 (50–133)	85 (61–142)	79 (50–133)	0.980
Plasma aspartate aminotransferase, AST (U/l)	89 (51.5–173.5)	89 (47–144)	100 (61–173.5)	0.618
Serum lipids				
Serum LDL cholesterol (mmol/l)	1.53 (1.38–2.01)	1.53 (1.17–1.72)	2.01 (1.73–2.41)	0.014
Serum total cholesterol, TC (mmol/l)	1.98 (1.89–2.66)	2.11 (1.74–2.49)	2.54 (2.28–2.78)	0.274
Serum triglycerides, TG (mmol/l)	0.66 (0.65–1.365)	0.66 (0.47–0.92)	1.33 (0.8–1.79)	0.019
Markers of cholestasis				
Plasma total bilirubin (*μ*mol/l)	20.9 (9.3–23.1)	16.15 (16.15–22.75.3)	12.85 (9–19.05)	0.492
Plasma conjugated bilirubin (*μ*mol/l)	8.5 (1.93–8.89)	8.5 (4.31–11.5)	2.5 (1.18–2.88)	0.045
