# Supplementary material for: p38α MAPK antagonizing JNK to control the hepatic fat accumulation in pediatric patients onset intestinal failure
Source: Cell Death Dis. 2017 Oct 12;8(10):e3110–. doi: 10.1038/cddis.2017.523 (PMC5682685; doi:10.1038/cddis.2017.523)
Supplement: Supplementary Information [file cddis2017523x1.docx]

**Supplemental data**

**p38α MAPK antagonizing JNK to control the hepatic fat accumulation in pediatric patients onset intestinal failure**

Yongtao Xiao, Jun Wang, Weihui Yan, Kejun Zhou, Yi Cao, Wei Cai

**Supplementary Materials and Methods**

**Supplementary Table 1-8**

**Supplementary Figure 1**

**Supplementary Reference**

**Supplementary Materials and Methods**

*Materials*

The main materials and reagents used in this study were showed following table.

| Reagents | Manufactures | Cat NO |
| --- | --- | --- |
| ACOX1 Rabbit Polyclonal antibody | proteintech | 10957-1-AP |
| CPT1A Rabbit Polyclonal antibody | proteintech | 15184-1-AP |
| Anti-PGC1 alpha antibody | Abcam | ab54481 |
| Anti-PPAR alpha antibody | Abcam | ab8934 |
| Phospho-p38 MAPK (Thr180/Tyr182) | Cell Signaling Technology | #4511 |
| Phospho-SAPK/JNK (Thr183/Tyr185) | Cell Signaling Technology | #4668 |
| Anti-FXR mouse monoantibody | Invitrogen | 417200 |
| Anti-Cyp7a1 Antibody | Millipore | MABD42 |
| EnzyChromTM Triglyceride Assay Kit | BioAssaySystems | ETGA-200 |
| EnzyChromTM Phospholipid Assay Kit | BioAssaySystems | EPLP-100 |
| SP600125 | Sigma-aldrich | S5567 |
| SB 203580 | Sigma-aldrich | S8307 |
| GW7647 | Sigma-aldrich | G6793-5mg |
| SYBR Master mix | Thermofisher scientific | 4385612 |
| High-Capacity cDNA Reverse Transcription Kit | Thermofisher scientific | 4368813 |
| HCS LipidTOX™ Green Neutral Lipid Stain | Thermofisher scientific | H34475 |

*Biochemical measurements in blood*

The human blood samples were analyzed for alanine aminotransferase (ALT), aspartate aminotransferase (AST), γ-glutamyl-transferase (AST) and bilirubin and conjugated bilirubin by using routine hospital laboratory methods. Serum total cholesterol (TC), triglycerides (TG), and low density lipoprotein cholesterol (LDL-C) were determined enzymatically. The plasma biochemistries for all animals were analysed with a CHEMIX-180 multiple analyser (Sysmex Corporation, Japan) as previously described [^1^](#_ENREF_1).

*Histological analyses and fibrosis determination*

### Histological examination was stained with hematoxylin and eosin (H&E). Fibrosis was determined by mason’s trichrome stain according to the method described in a previous study [^2^](#_ENREF_2). Liver biopsies were analyzed by two researchers and a pathologist, blinded to clinical data, until consensus was reached. Liver tissues were analyzed for apoptosis using TdT-mediated dUTP nick-end labeling (TUNEL) staining.

### *Transmission electron microscopy (TEM)*

The liver samples from patients were fixed with 3% glutaraldehyde at room temperature. The tissues were then washed and postfixed with 1% osmium tetroxide in 0.05 mol/L sodium cacodylate buffer (pH 7.4) at 4 °C for 2 hours and stained with saturated uranyl acetate for 3.5 hours at room temperature, dehydrated in graded alcohol and embedded in Eponate 12 resin (Ted Pella, Inc., United States). Sections were then cut with a diamond knife, stained with a saturated solution of uranyl acetate in 50% ethanol and lead citrate. Sections were examined and photographed with a Philips CM120 electron microscope at 80 KV.

*Fatty acid preparation*

Fatty acids palmitate (PA) (Sigma-Aldrich ) were dissolved in 0.1 M sodium hydroxide (final concentration 100 mM) at 65 °C for 2-4 h and then complexed with 10% fatty acid-free bovine serum albumin (BSA).

*Promoters of luciferase reporter*

To construct reporter vectors carrying promoters of CYP7A1, PPARA and PPARGC1A, we synthesized the fragments containing the promoters for human CYP7A1,PPARA and PPARGC1A that were showed below, and cloned them into the psiCHECK2 luciferase vector (Promega, Madison, WI).

Name=PPARA; Entrez_ID=5465; Genome=hg18; chr22+:44924124-44925451; TSS=44925163; Upstream=1039, Downstream=288; Length=1328;

AGTTGTTTGTTTTGTTTTGTTGAGACACGGTCTTACTCTGTCACCCAGGCTGGAGTGCAGTGGTGTGATCTTGGCTCACTCCTGGCCTCAAGTGATCCACCCACCTCAGCCTCCTAAGTAGCTGGGACCACAGGTGTGTACCACCACACCCAGCTAATTTTTTTTTTTTTTTTTTTTTTTTTTGTAGGGACGGGGTTTTGTCATATCACCCAGGCTGGTCTCAAACTCCTGGACTCAAGGGATCAGCCTGTCTCAGCCTCCCAAAGTGCTGGGATTACAGGTGTGAGCCACTGCACCGGTCCTGATTTGAGTTTTTGTAAGACAGGGAACAATGTTCAGAATTTAGCACCAATGTCAGACTCATTCTGTAAATTTTTATTGAACGTCTGCCTGGTGTAGGAGAGGAAGATGACAGACAAGAATTCTTCCTCCAAGAGTTACAGGTCAGTTGAGCAGAAAAGGCATACATCAATACCCACAATGAGAGTTGTCGTGATTCAGAGGAGGGACAAAGTCCTTCCCCTGGAGGGATCCTGAGCACTTTGGAGAGGAAAGGCATCTGTACTGCCCCCCAAATGTGTAGAATGGGATGCATTCCTGGCAGAAAGAAGTAGGATAAAGTACAGAGGCCAGGGCTGGGTGCAGTGGTTCACGCCTGTAATCCCAGCACTTTGGGAGGCCGAGACAGCAGATCACCTGAGGTCAGGAGTTCGAGACCAGCCTGGTCAACATGGCAAAACCCTCTCTCTACTAAAAATACAAAAATTAGCCAGGCACAATGGCAGGTACCTGTAATCCCAGCTACTTGGGAGGCTGAGGCAGGAGAATTGCTTGAGCCCAGGAGGCAGAGATCGCAGTGAGCCAAGACTGCGCCACTGCACTCCAGCCTGGGCAACAGAGCAAGACTCTGTCTCATAAAAAAAGAAAAAAAAAAAGTACAGAGTCCAGGAAGCCTGGGGTGGGGCTGGCAGATGCCGAGTCATCTATTTTGGCCAGAGTTCAAGGCTTGCTAGGGGACATGAAGAGAAGATTCGTGCATTCTAGTTCAAACTCCACCAGATATTTGAGCTCCTTCTCTGTACCAGGCATTGTTCTAAGATACGTAAGTGAACAAAACCCATGACACCCTCGTCTATGAGAGCTGATCCTCTGGCAGGGACAGACAGGTCATGAGTGGAGTGATGGAGCAGCTGGCCTGGTGACTTAGCCGCCTTCAGGTACAGTAGGAGGAGCAAGCCCAGGACAGGTGAGTGGGTCAAGGGTGCCAGAAGGGGTGAGGGCACCAGGAAGCTGGTCCAGTTTGGCTTCCCTGAGGTGGTGACCAGGACCTAGCATCTG

Name=PPARGC1A; Entrez_ID=10891; Genome=hg38; chr4-:23891527-23889956; TSS=23890077; Upstream=1450, Downstream=121; Length=1572; TGTGTCCAGTACCTTGAGTTTGTTATGTATTCAATATGTAGTGTCATCATAAAACAGTTGCACCTACCTGCATTAGCCCTCATTGTCTCAAGGTACAAGCTGAAAAATAATAGAAAGTAAAGCTCAGGAATTGAATATTTCTGCTAATAGTGTGTTGGTATTTTTCCCTCAGTTCACAGACATTCTTGATTTCAAAACGCAAACTACACAACCCAGGGCACTAGGGTTGGAATTCAATGTTTATTCAAAAAGGCACCCTAAGGCAGTTAGGGAGGAAACGCTACATGTATGAAAAATAGGAGCCGGGAATCAAAGCTGATCTGAGCAGAGCAGCAGCGACTGTATTTACTAACACTTGTTTTCTGGGAGCCTATGAGAGAAATGGAAATAATTAGAAGGAAGCTGAAAGGATGGGGTTTTGTGGCTTGTTCTCCTTATATGGAGCAAAGAAAACTGCAGCAACTCTTCGGGAGCTGGTATTCCCTACTGCCATGGGGGCAGCCGAATTCTGGGTGGAGGAGTTTGTTTATACCTTAACACATACAGGCTATTTTGTTGATTAAACAAGCAAAAAAAAAAAAAAAAAAAAAAAAAGCCCCGTTTGCGCTTTCAAACACTCCCTCAATGAGAAAATGTCTCATAAAAATGCATCATGTGATAAGCTCTTGCTTTAGTCCCAAACTGAGCTTGAGTCCACTTGGAGATCTTAGAATTAAAGAGTTCTTAGGGAATACACGTTTTAGCTAAGAATATAGTTACTCTGTCATGAAACAGGGAGCTTTGCCACTTGCTTGTTTTGGAAGGAAAATAAATTAAAAAAAGATTGCAGGGGATTTTGGTTATTATATGGCCAGGGCTCCGTTTAGAGTCTGTGGCATTCAAAGCTGGCTTTAATCACAGCATGATGCTTGAAGCCTCCAAAAGTCTAAGTGTTTCCTTTCTTTCTTTCTTTTCTTTTTCTTTCTTTTTTTTTTTTTTTAAAGCGTTACTTCACTGAAGCAGAGGGCTGCCTTTGAGTGACGTCACGAGTTAGAGCAGCAAGCTGCACAGGGGAAGGGAGGCTGGGTGAGTGACAGCCCAGCCTACTTTTTAATAGCTTTGTCATGTGACTGGGGACTGTAGTAAGACAGGTGCCTTCAGTTCACTCTCAGTAAGGGGCTGGTTGCCTGCATGAGTGTGTGCTCTGTGTCACTGTGGATTGGAGTTGAAAAAGCTTGACTGGCGTCATTCAGGAGCTGG

Name=CYP7A1;Entrez_ID=1581;Genome=hg38;chr8-:58501624-58500148;TSS=58500161;Upstream=1463,Downstream=13;Length=1477;

AGCCAAAACTGGAAACAACCCACATATCCATCAATAGGAAATCAGTTAAATAAATTATAGTACATTTATCCAATGGAAGATTAAGCACATATTCAATATAATTATTTATACACACATATAGATACACACATGTATAAATATAGAGAATACTGTGGGTGTATGTGTGTGTGTGTTTATATACATATATATACACACACAGTACTGTTGCCTACCTTCTTTTGTCTTAATTCTGTGAACTCTCATTCACTCTGCTTCAGTAGGATACATCCTTCTTTTTGGTTCTTAGACTCACCAAGTTGATCCTTGACTCAAGACATTGCATTTGCTGCTTCCTCTTCCTGGAATATCCTTCCTTCTGATATTCACATGAGTAGTCTCTTCTTGTCATTCAGATCTCAAATGTCACAATTTCAGAGAGCCCATCTCTGATCATCATATCTAAAGTTGTCCTCATTCCCCCATAGCTTTCTATACCATGTTTTATTTTTTTCATAACATGTATTTTATTACTCCTTTCTCCATTGGAATAGAATCTCCATTAGATTAGGAAATCTGCCTATCTTATTAATGCCTGCAACTGGAATACTTTTGAAGAGTTCTTGGCACGTAATAAATACTCAACTAATATTTTTGTGTACACAGAAATAAAGTTTGGAAGAACAGATGCCAAATTGTTACTAGTGGTTACTTCTGAGTAAAGGAGTAGCATGGTAGGTAAATTATTAATAGATGTTCACTTTCCACCAAGATATGTTTTAGTTAGTCTTAACTTACTTGAAATGAAATTTATTACTTTAATAATTAGAAACATTGATAAACATTTTAGTCACAAGAATGATAGATAAAATTTTGATGCTTCCAATAAGTTATATTTATCTAGAGGATGCACTTATGTAGAATACTCTCTTGAGGATGTTAGGTGAGTAACATGTTACTATATGTAGTAAAATATCTATGATTTTATAAAAGCACTGAAACATGAAGCAGCAGAAATGTTTTTCCCAGTTCTCTTTCCTCTGAACTTGATCACCGTCTCTCTGGCAAAGCACCTAAATTAATTCTTCTTTAAAAGTTAACAAGACCAAATTATAAGCTTGATGAATAACTCATTCTTATCTTTCTTTAAATGATTATAGTTTATGTATTTATTAGCTATGCCCATCTTAAACAGGTTTATTTGTTCTTTTTACACATACCAAACTCTTAATATTAGCTGTTGTCCCCAGGTCCGAATGTTAAGTCAACATATATTTGAGAGACCTTCAACTTATCAAGTATTGCAGGTCTCTGATTGCTTTGGAACCACTTCTGATACCTGTGGACTTAGTTCAAGGCCAGTTACTACCACTTTTTTTTTTCTAATAGAATGAACAAATGGCTAATTGTTTGCTTTGTCAACCAAGCTCAAGTTAATGGATCTGGATACTATGTATATAAAAAGCCTAGCTTGAGTCTCTTTTCAGTGGCATCCTTCCCT

*Oil red O staining*

Oil red O staining of liver sections was adopted to the method as previously described [^3^](#_ENREF_3).

*Bile Acids Measurements*

Bile acids in the plasma and liver were measured according to the previously reported method [^4^](#_ENREF_4)^,^ [^5^](#_ENREF_5). The detection was performed with a Waters ACQUITY ultra performance liquid chromatography (BEH C18 1.7 μm 2.1×100mm column) coupled with Waters Xevo TQ-S triple quadrupole mass spectrometry. Data acquisition and bile acids quantification were performed using the MassLynx 4.1 software (Waters). The bile acid inculded the cholic acid (CA), glycocholic acid (GCA), taurocholic acid ( TCA), chenodeoxycholic acid (CDCA), glycochenodeoxycholic acid (GCDCA), taurochenodeoxycholic acid (TCDCA), deoxycholic acid (DCA), glycodeoxycholic acid (GDCA), taurodeoxycholic acid (TDCA), ursodeoxycholic acid (UDCA), glycoursodeoxycholic acid (GUDCA), tauroursodeoxycholic acid (TUDCA), lithocholic acid (LCA), glycolithocholic acid (GLCA), taurolithocholic acid ( TLCA), hyocholic acid (HCA), glycohyocholic acid (GHCA), taurohyocholic acid (THCA), α-muricholic acid (αMCA), tauro-α-muricholic acid (TαMCA), β-muricholic acid (βMCA), tauro- β-muricholic acid ( TβMCA), ω-muricholic acid (ωMCA), tauro-ω-muricholic acid (TωMCA), hyodeoxycholic acid (HDCA), glycohyodeoxycholic acid (GHDCA), taurohyodeoxycholi acid (THDCA), murocholic acid (MuroCA), dehydrocholic acid (DHCA), glycodehydrocholic acid (GDHCA), taurodehydrocholic acid (TDHCA), 3-dehydrocholic acid (3-DHCA), 7-dehydrocholic acid (7-DHCA), isodeoxycholic acid (isoDCA), apocholic acid (apoCA), 6-ketolithocholic acid (6-KLCA), 7-ketolithocholic acid (7-KLCA), 12-ketolithocholic acid (12-KLCA), 23-nordeoxycholic acid (23norDCA), dehydrolithocholic acid (DHLCA). Deuterated internal standards (IS) lithocholic acid-2,2,4,4-D4 (LCA-D4) and cholic acid-2,2,4,4-D4 (CA-D4) and Chenodeoxycholic Acid 24-Acyl-β-D-glucuronide (CDCA-24G).

**Supplementary Table 1: The components of PN**

| Component | Volume (mL) |
| --- | --- |
| 8.5% amino acids | 30 |
| MCT/LCT emulsion | 20.2 |
| 50% glucose | 40 |
| addamel | 0.2 |
| lipid-soluble vitamin | 0.2 |
| water-soluble vitamins | 0.2 |
| 10% sodium chloride | 3 |
| 10% potassium chloride | 2 |
| 10% calcium gluconate | 2 |
| phosphorus | 0.2 |

LCT, long chain triglyceride; MCT medium chain triglyceride; 1.33 kcal/mL; Total 98 mL (130.2 kcal); 30 mL/day

**Supplementary Table 2: The sequences of siRNAs**

| MAPK14 siRNAs duplexes pool | | | |
| --- | --- | --- | --- |
|  | siRNA-1 | sense（5'-3'） | GGCACACAGAUGAUGAAAUTT |
|  |  | antisense（5'-3'） | AUUUCAUCAUCUGUGUGCCTT |
|  | siRNA-2 | sense（5'-3'） | GGGCAGAUCUGAACAACAUTT |
|  |  | antisense（5'-3'） | AUGUUGUUCAGAUCUGCCCTT |
|  | siRNA-3 | sense（5'-3'） | CCGAGGUCUAAAGUAUAUATT |
|  |  | antisense（5'-3'） | UAUAUACUUUAGACCUCGGTT |
| MAPK8 siRNAs duplexes pool | | | |
|  | siRNA-1 | sense（5'-3'） | GCCCAGUAAUAUAGUAGUATT |
|  |  | antisense（5'-3'） | UACUACUAUAUUACUGGGCTT |
|  | siRNA-2 | sense（5'-3'） | GCCGACCAUUUCAGAAUCATT |
|  |  | antisense（5'-3'） | UGAUUCUGAAAUGGUCGGCTT |
|  | siRNA-3 | sense（5'-3'） | GCUGGUAAUAGAUGCAUCUTT |
|  |  | antisense（5'-3'） | AGAUGCAUCUAUUACCAGCTT |
| MAPK9 siRNAs duplexes pool | | | |
|  | siRNA-1 | sense（5'-3'） | GUGAACUUGUCCUCUUAAATT |
|  |  | antisense（5'-3'） | UUUAAGAGGACAAGUUCACTT |
|  | siRNA-2 | sense（5'-3'） | CGUGCACUAACUUCAUGAUTT |
|  |  | antisense（5'-3'） | AUCAUGAAGUUAGUGCACGTT |
|  | siRNA-3 | sense（5'-3'） | GGUGAAAGGUUGUGUGAUATT |
|  |  | antisense（5'-3'） | UAUCACACAACCUUUCACCTT |
| MKK3 siRNAs duplexes pool | | | |
|  | siRNA-1 | sense（5'-3'） | CAUCCUUGGACAAGUUCUATT |
|  |  | antisense（5'-3'） | UAGAACUUGUCCAAGGAUGTT |
|  | siRNA-2 | sense（5'-3'） | GGAAGAAGGAUCUACGGAUTT |
|  |  | antisense（5'-3'） | AUCCGUAGAUCCUUCUUCCTT |
|  | siRNA-3 | sense（5'-3'） | GCUGAUGACUUGGUGACCATT |
|  |  | antisense（5'-3'） | UGGUCACCAAGUCAUCAGCTT |
| MMK6 siRNAs duplexes pool | | | |
|  | siRNA-1 | sense（5'-3'） | GGUGGACUCUGUUGCUAAATT |
|  |  | antisense（5'-3'） | UUUAGCAACAGAGUCCACCTT |
|  | siRNA-2 | sense（5'-3'） | CACACCACCUCGAGAUUUATT |
|  |  | antisense（5'-3'） | UAAAUCUCGAGGUGGUGUGTT |
|  | siRNA-3 | sense（5'-3'） | GGAUACAUCACUAGAUAAATT |
|  |  | antisense（5'-3'） | UUUAUCUAGUGAUGUAUCCTT |
| MKK4 siRNAs duplexes pool | | | |
|  | siRNA-1 | sense（5'-3'） | GUCGCAUGCUAUGUUUGUATT |
|  |  | antisense（5'-3'） | UACAAACAUAGCAUGCGACTT |
|  | siRNA-2 | sense（5'-3'） | CACAGGCCGAUUUCCUUAUTT |
|  |  | antisense（5'-3'） | AUAAGGAAAUCGGCCUGUGTT |
|  | siRNA-3 | sense（5'-3'） | CGCAUCACGACAAGGAUAUTT |
|  |  | antisense（5'-3'） | AUAUCCUUGUCGUGAUGCGTT |
|  | MKK7 siRNAs duplexes pool | | |
|  | siRNA-1 | sense（5'-3'） | CAGGACAGUUUCCCUACAATT |
|  |  | antisense（5'-3'） | UUGUAGGGAAACUGUCCUGTT |
|  | siRNA-2 | sense（5'-3'） | GCAAGAUGACAGUGGCGAUTT |
|  |  | antisense（5'-3'） | AUCGCCACUGUCAUCUUGCTT |
|  | siRNA-3 | sense（5'-3'） | GGAAGAGACCAAAGUAUAATT |
|  |  | antisense（5'-3'） | UUAUACUUUGGUCUCUUCCTT |
| PPARGC1A siRNAs duplexes pool | | | |
|  | siRNA-1 | sense（5'-3'） | GAAGAGCCGUCUCUACUUATT |
|  |  | antisense（5'-3'） | UAAGUAGAGACGGCUCUUCTT |
|  | siRNA-2 | sense（5'-3'） | CCAAGACUCUAGACAACUATT |
|  |  | antisense（5'-3'） | UAGUUGUCUAGAGUCUUGGTT |
|  | siRNA-3 | sense（5'-3'） | CCAUAUUCCAGGUCAAGAUTT |
|  |  | antisense（5'-3'） | AUCUUGACCUGGAAUAUGGTT |
|  |  |  |  |

**Supplementary Table 3: The sequences of primers**

| Species | Genes |  | Sequences |
| --- | --- | --- | --- |
| Human | Actin | Forward （5'-3'） | CTTTGCAGCTCCTTCGTTGC |
|  |  | Reverse（5'-3'） | CCTTCTGACCCATTCCCACC |
| Human | CYP7A1 | Forward （5'-3'） | GCTCTTTACCCACAGTTAATGC |
|  |  | Reverse（5'-3'） | TTGTCTTCCCGTTTTCATCA |
| Human | MAPK14 | Forward （5'-3'） | GAGAGGCCCACGTTCTACC |
|  |  | Reverse（5'-3'） | CGTAACCCCGTTTTTGTGTCA |
| Human | MAPK8 | Forward （5'-3'） | CTGAAGCAGAAGCTCCACCA |
|  |  | Reverse（5'-3'） | CCTGTGCTAAAGGAGAGGGC |
| Human | MAPK9 | Forward （5'-3'） | AGTGGGTTGCATCATGGGAG |
|  |  | Reverse（5'-3'） | TCTGCTGATGGTGTTCCCAG |
| Human | PPARα | Forward （5'-3'） | ATGGTGGACACGGAAAGCC |
|  |  | Reverse（5'-3'） | CGATGGATTGCGAAATCTCTTGG |
| Human | PPARGC1A | Forward （5'-3'） | TGCATGAGTGTGTGCTCTGT |
|  |  | Reverse（5'-3'） | GCACACTCGATGTCACTCCA |
| Human | FXR | Forward （5'-3'） | GGAAATGGCAACCAATCA |
|  |  | Reverse（5'-3'） | TAGCTTCAACCGCAGACC |
| Human | CPT1A | Forward （5'-3'） | TCCAGTTGGCTTATCGTGGTG |
|  |  | Reverse（5'-3'） | TCCAGAGTCCGATTGATTTTTGC |
| Human | ACOX1 | Forward （5'-3'） | ACTCGCAGCCAGCGTTATG |
|  |  | Reverse（5'-3'） | AGGGTCAGCGATGCCAAAC |
| Rat | 18S | Forward （5'-3'） | ACGGACCAGAGCGAAAGCAT |
|  |  | Reverse（5'-3'） | TGTCAATCCTGTCCGTGTCC |
| Rat | CYP7A1 | Forward （5'-3'） | TGAAAGCGGGAAAGCAAAGACCAC |
|  |  | Reverse（5'-3'） | TCTTGGACGGCAAAGAGTCTTCCA |
| Rat | Mapk14 | Forward （5'-3'） | TGGATATTTGGTCCGTGGGC |
|  |  | Reverse（5'-3'） | CCCCGTCAGACGCATTATCT |
| Rat | Mapk8 | Forward （5'-3'） | TCCAGTTCTCGTACCCGCTA |
|  |  | Reverse（5'-3'） | AGCATGGCGTGACACAGTAA |
| Rat | Mapk9 | Forward （5'-3'） | CTGTTTGGTATGACCCCGCT |
|  |  | Reverse（5'-3'） | TGCTGCTTACTGCTGCATCT |
| Rat | PPARα | Forward （5'-3'） | GTCCTCTGGTTGTCCCCTTG |
|  |  | Reverse（5'-3'） | GTCAGTTCACAGGGAAGGCA |
| Rat | Ppargc1a | Forward （5'-3'） | TTCAGGAGCTGGATGGCTTG |
|  |  | Reverse（5'-3'） | TATGTTCGCGGGCTCATTGT |
| Rat | CPT1A | Forward （5'-3'） | CCACGAAGCCCTCAAACAGA |
|  |  | Reverse（5'-3'） | GGTCCGACTGATCTTTGCGA |
| Rat | ACOX1 | Forward （5'-3'） | CTCACTCGAAGCCAGCGTTA |
|  |  | Reverse（5'-3'） | TTGAGGCCAACAGGTTCCAC |
| Rat | FXR | Forward （5'-3'） | CCA ACCTGGGTTTCTACC C |
|  |  | Reverse（5'-3'） | CACACAGCTCATCCCCTT T |

**Supplementary Table 4: The profiles of bile acid in patients’ liver (nmol/mg protein)**

|  | Without Steatosis (n=9) | | With Steatosis (n=7) | |
| --- | --- | --- | --- | --- |
|  | mean | SD | mean | SD |
| Primary BA | | | | |
| CA | 2.46 | 1.76 | 0.65 | 0.86 |
| CDCA | 0.69 | 0.97 | 0.08 | 0.11 |
| Secondary BA | | | | |
| TCA | 77.63 | 37.26 | 90.34 | 41.35 |
| GCA | 86.58 | 60.14 | 78.73 | 26.75 |
| DCA | 1.15 | 2.02 | 0.50 | 0.26 |
| TCDCA | 45.61 | 38.67 | 36.37 | 18.20 |
| GCDCA | 85.81 | 42.68 | 75.33 | 29.74 |
| UDCA | 0.07 | 0.06 | 0.05 | 0.05 |
| TUDCA | 0.49 | 0.76 | 0.21 | 0.17 |
| GUDCA | 5.96 | 10.51 | 1.38 | 3.03 |

**Supplementary Table 5: The profiles of bile acid in patients’ serum (nmol/L)**

|  | Without Steatosis(n=12) | | With Steatosis (n=9) | |
| --- | --- | --- | --- | --- |
|  | mean | SD | mean | SD |
| Primary BA | | | | |
| CA | 863.30 | 1252.67 | 3484.32 | 5508.43 |
| CDCA | 571.57 | 629.21 | 1479.46 | 2861.36 |
| Secondary BA | | | | |
| LCA | 1.99 | 1.92 | 1.18 | 0.99 |
| 6ketoLCA | 2.65 | 2.78 | 1.10 | 0.90 |
| 7ketoLCA | 9.55 | 7.69 | 21.03 | 31.27 |
| 12ketoLCA | 3.20 | 2.12 | 6.08 | 8.56 |
| apoCA | 16.31 | 22.16 | 39.62 | 69.12 |
| muroCA | 62.54 | 94.46 | 37.97 | 70.76 |
| UDCA | 165.45 | 347.11 | 130.79 | 151.65 |
| HDCA | 0.84 | 0.67 | 0.73 | 0.31 |
| DCA | 23.43 | 36.08 | 24.13 | 34.79 |
| isoDCA | 1.48 | 0.14 | 1.52 | 0.12 |
| 7DHCA | 138.83 | 435.03 | 560.97 | 737.87 |
| 3DHCA | 26.02 | 34.50 | 95.48 | 195.63 |
| HCA | 66.49 | 146.41 | 42.06 | 74.20 |
| GLCA | 2.53 | 4.92 | 1.40 | 2.19 |
| GUDCA | 500.03 | 1072.60 | 193.64 | 415.43 |
| GHDCA | 2.01 | 4.44 | 0.53 | 0.05 |
| GCDCA | 1328.63 | 1038.66 | 442.37 | 660.60 |
| GDCA | 280.94 | 660.06 | 35.30 | 48.79 |
| GHCA | 154.90 | 385.14 | 74.68 | 116.26 |
| GCA | 909.13 | 1540.18 | 1402.75 | 2853.65 |
| TLCA | 1.75 | 2.50 | 1.00 | 1.27 |
| TUDCA | 18.36 | 44.48 | 1.29 | 1.41 |
| TCDCA | 479.46 | 939.10 | 31.28 | 32.68 |
| TDCA | 61.08 | 156.76 | 3.61 | 5.13 |
| LCA3S | 4.57 | 6.99 | 5.71 | 8.60 |
| CDCA24G | 17.70 | 18.54 | 51.81 | 73.48 |
| TωMCA | 123.67 | 200.65 | 0.20 |  |
| TαMCA | 129.37 | 430.41 | 5.41 | 0.78 |
| TβMCA | 0.23 | 0.30 | 0.30 | 0.16 |
| THCA | 88.24 | 209.66 | 1.54 | 1.98 |
| TCA | 185.18 | 247.22 | 62.65 | 83.96 |

**Supplementary Table 6: The biochemical measurements in rat’s serum**

|  | Sham | | PN | | PN+SB203580 | | PN+SP600125 | |
| --- | --- | --- | --- | --- | --- | --- | --- | --- |
|  | Mean | SD | Mean | SD | Mean | SD | Mean | SD |
| Total cholesterol, TC (mmol/L) | 1.16 | 0.22 | 1.45 | 0.38 | 1.57 | 0.45 | 1.62 | 0.34 |
| Triglycerides, TG (mmol/L) | 0.49 | 0.22 | 0.59 | 0.28 | 0.65 | 0.26 | 0.63 | 0.12 |
| Albumin, ALB (g/L) | 21.99 | 1.91 | 14.25 | 2.54 | 14.80 | 2.56 | 15.95 | 3.90 |
| Alkaline phosphatase, ALP (U/L) | 374.25 | 72.61 | 425.10 | 155.80 | 383.05 | 122.52 | 460.85 | 157.84 |
| Alanine aminotransferase, ALT（(U/L) | 72.21 | 15.22 | 44.25 | 23.55 | 62.55 | 50.65 | 64.90 | 36.10 |
| Aspartate aminotransferase, AST(U/L) | 213.96 | 133.01 | 177.10 | 65.06 | 196.95 | 117.63 | 171.90 | 90.92 |
| Conjugated bilirubin(μmol/L) | 7.13 | 0.94 | 7.97 | 0.86 | 7.52 | 1.87 | 7.72 | 0.51 |
| Total bilirubin(μmol/L) | 5.10 | 1.09 | 7.35 | 2.38 | 6.30 | 3.46 | 9.15 | 2.23 |
| Glucose (mmol/L) | 11.78 | 5.26 | 8.76 | 1.52 | 6.78 | 3.95 | 7.46 | 0.66 |

**Supplementary Table 7: The profiles of bile acid in rat’s liver (nmol/mg protein)**

|  | Sham | | PN | | PN+SB203580 | | PN+SP600125 | |
| --- | --- | --- | --- | --- | --- | --- | --- | --- |
|  | mean | SD | mean | SD | mean | SD | mean | SD |
| Primary BA |  |  |  |  |  |  |  |  |
| ωMCA | 14.22 | 12.52 | 2.98 | 2.26 | 2.00 | 1.80 | 2.06 | 2.54 |
| αMCA | 16.09 | 10.78 | 1.11 | 0.63 | 1.85 | 2.72 | 3.88 | 5.29 |
| βMCA | 39.04 | 21.65 | 7.89 | 5.64 | 6.40 | 4.83 | 22.07 | 32.67 |
| CA | 62.71 | 60.51 | 14.89 | 17.88 | 9.35 | 6.70 | 20.86 | 28.51 |
| CDCA | 5.01 | 2.81 | 0.70 | 0.50 | 1.00 | 1.30 | 1.88 | 2.28 |
| Secondary BA |  |  |  |  |  |  |  |  |
| LCA | 0.93 | 0.69 | 0.12 | 0.11 | 0.14 | 0.13 | 0.20 | 0.20 |
| 6ketoLCA | 0.65 | 0.76 | 0.33 | 0.52 | 0.08 | 0.10 | 0.28 | 0.48 |
| 7ketoLCA | 0.08 | 0.07 | 0.04 | 0.04 | 0.03 | 0.02 | 0.05 | 0.05 |
| 12ketoLCA | 0.03 | 0.06 | 0.00 | 0.01 | 0.02 | 0.04 | 0.05 | 0.10 |
| apoCA | 0.08 | 0.10 | 0.05 | 0.11 | 0.05 | 0.07 | 0.01 | 0.02 |
| muroCA | 5.75 | 4.96 | 0.98 | 0.70 | 0.43 | 0.57 | 0.82 | 1.26 |
| UDCA | 6.31 | 3.83 | 0.46 | 0.23 | 0.73 | 0.87 | 3.08 | 4.51 |
| HDCA | 29.78 | 22.27 | 7.25 | 4.51 | 3.11 | 2.31 | 10.69 | 16.06 |
| DCA | 7.40 | 15.32 | 1.06 | 1.29 | 0.34 | 0.21 | 0.46 | 0.65 |
| isoDCA | 0.03 | 0.01 | 0.04 | 0.03 | 0.03 | 0.02 | 0.04 | 0.01 |
| 7DHCA | 37.98 | 31.24 | 2.54 | 1.58 | 2.46 | 3.65 | 4.72 | 6.42 |
| 3DHCA | 0.22 | 0.34 | 0.10 | 0.15 | 0.08 | 0.07 | 0.08 | 0.10 |
| HCA | 1.02 | 1.04 | 0.09 | 0.06 | 0.08 | 0.09 | 0.15 | 0.19 |
| GLCA | 3.70 | 1.45 | 0.83 | 0.69 | 0.34 | 0.32 | 0.18 | 0.15 |
| GUDCA | 32.79 | 11.98 | 5.53 | 3.56 | 4.88 | 3.55 | 8.38 | 6.93 |
| GHDCA | 97.31 | 28.26 | 93.41 | 82.39 | 38.67 | 41.03 | 39.83 | 36.57 |
| GCDCA | 121.48 | 42.29 | 35.45 | 23.92 | 23.88 | 19.07 | 30.48 | 9.38 |
| GDCA | 120.56 | 121.97 | 37.73 | 35.39 | 15.83 | 18.65 | 8.68 | 2.78 |
| GHCA | 2.63 | 1.69 | 0.89 | 0.93 | 0.71 | 1.11 | 0.56 | 0.34 |
| GCA | 309.93 | 83.97 | 110.72 | 61.32 | 77.15 | 55.24 | 131.42 | 87.83 |
| TLCA | 2.65 | 1.09 | 1.53 | 1.61 | 0.62 | 0.30 | 0.52 | 0.29 |
| TUDCA | 24.39 | 9.28 | 39.89 | 25.81 | 20.89 | 15.40 | 30.74 | 20.71 |
| TCDCA | 65.09 | 21.36 | 33.48 | 23.73 | 27.37 | 12.06 | 59.20 | 20.24 |
| TDCA | 68.77 | 102.69 | 29.41 | 33.79 | 12.64 | 9.52 | 13.31 | 3.41 |
| LCA3S | 0.10 | 0.06 | 0.02 | 0.02 | 0.02 | 0.03 | 0.01 | 0.02 |
| CDCA24G | 0.41 | 0.27 | 0.07 | 0.06 | 0.06 | 0.05 | 0.09 | 0.04 |
| TωMCA | 11.24 | 8.45 | 4.16 | 3.29 | 0.81 | 0.90 | 1.40 | 1.83 |
| TαMCA | 43.20 | 17.06 | 15.32 | 8.73 | 16.73 | 14.52 | 35.27 | 25.15 |
| TβMCA | 46.00 | 9.34 | 83.79 | 118.85 | 64.09 | 39.97 | 122.19 | 67.19 |
| THCA | 0.72 | 0.82 | 0.15 | 0.14 | 0.30 | 0.37 | 0.41 | 0.37 |
| TCA | 243.80 | 62.00 | 177.64 | 120.39 | 162.79 | 62.18 | 300.68 | 111.47 |

**Supplementary Table 8: The profiles of bile acid in rat’s serum (nmol/L)**

|  | Sham | | PN | | PN+SB203580 | | PN+SP600125 | |
| --- | --- | --- | --- | --- | --- | --- | --- | --- |
|  | mean | SD | mean | SD | mean | SD | mean | SD |
| Primary BA | | | | | | | | |
| wMCA | 2821.52 | 2024.01 | 1333.88 | 570.39 | 700.85 | 246.68 | 1225.87 | 373.57 |
| aMCA | 7327.13 | 5503.55 | 1697.38 | 529.08 | 1144.42 | 746.03 | 3376.67 | 1421.4 |
| bMCA | 5109.18 | 3593.73 | 1984.72 | 872.78 | 1252.78 | 496.89 | 4337.23 | 3659.7 |
| CA | 20438.4 | 12524.80 | 12734.63 | 6627.12 | 6098.17 | 3549.1 | 13406.8 | 5614.8 |
| CDCA | 7417.09 | 5576.82 | 1102.32 | 520.72 | 597.92 | 377.24 | 1728.02 | 732.78 |
| Secondary BA | | | | | | | | |
| LCA | 53.26 | 20.52 | 6.94 | 3.63 | 4.12 | 1.88 | 4.98 | 2.75 |
| 6ketoLCA | 213.15 | 188.91 | 146.10 | 49.73 | 96.33 | 96.70 | 109.23 | 64.87 |
| 7ketoLCA | 309.62 | 275.06 | 31.95 | 14.44 | 16.57 | 10.55 | 98.00 | 66.95 |
| 12ketoLCA | 10.87 | 6.41 | 5.25 | 3.04 | 3.00 | 2.10 | 52.85 | 5.44 |
| apoCA | 221.26 | 162.55 | 132.03 | 172.28 | 80.73 | 95.30 |  |  |
| muroCA | 496.14 | 414.31 | 236.27 | 98.26 | 89.08 | 59.17 | 143.42 | 113.91 |
| UDCA | 1674.97 | 1245.79 | 218.88 | 59.66 | 167.18 | 121.88 | 692.13 | 756.73 |
| HDCA | 8043.19 | 6664.9 | 5666.3 | 2342.4 | 2987.3 | 2804.9 | 3747.7 | 3234.1 |
|  |  |  |  |  |  |  |  |  |
| DCA | 1761.16 | 1464.43 | 935.75 | 521.34 | 213.77 | 215.61 | 333.70 | 47.46 |
| isoDCA | 2.15 | 0.74 | 1.97 | 0.98 | 1.58 | 0.29 | 1.90 | 0.9 |
| 7DHCA | 3365.40 | 2482.94 | 1231.57 | 964.55 | 387.93 | 335.47 | 1147.17 | 534.96 |
| 3DHCA | 350.68 | 270.06 | 151.05 | 92.00 | 60.53 | 34.54 | 127.85 | 77.66 |
| HCA | 273.09 | 225.98 | 89.23 | 65.55 | 74.12 | 87.94 | 112.45 | 45.56 |
| GLCA | 11.53 | 7.63 | 5.60 | 3.75 | 2.00 | 1.14 | 0.95 | 0.36 |
| GUDCA | 51.46 | 35.98 | 12.43 | 12.75 | 18.25 | 12.35 | 25.25 | 10.39 |
| GHDCA | 758.46 | 531.94 | 577.30 | 580.88 | 760.10 | 867.79 | 388.57 | 268.23 |
| GCDCA | 810.25 | 531.98 | 190.85 | 76.16 | 244.77 | 144.3 | 287.18 | 67.78 |
| GDCA | 284.55 | 203.72 | 195.88 | 207.66 | 139.57 | 177.1 | 42.38 | 15.90 |
| GHCA | 10.04 | 6.61 | 2.80 | 2.43 | 7.78 | 8.10 | 4.32 | 2.55 |
| GCA | 1141.89 | 562.90 | 669.62 | 660.39 | 424.22 | 246.5 | 852.88 | 411.73 |
| TLCA | 1.52 | 0.93 | 3.62 | 3.44 | 1.80 | 1.23 | 1.83 | 0.73 |
| TUDCA | 28.42 | 10.53 | 178.85 | 335.22 | 114.82 | 98.33 | 99.55 | 30.63 |
| TCDCA | 58.86 | 41.20 | 199.52 | 418.00 | 76.10 | 53.81 | 250.53 | 103.40 |
| TDCA | 17.63 | 16.07 | 61.05 | 80.42 | 32.60 | 39.60 | 42.78 | 20.72 |
| LCA3S | 4.25 | 2.33 | 6.30 | 2.08 | 1.85 | 0.34 | 1.83 | 0.54 |
| CDCA24G | 38.74 | 28.69 | 7.54 | 4.84 | 2.38 | 1.14 | 5.72 | 0.95 |
| TwMCA | 18.76 | 21.78 | 97.00 | 162.90 | 14.94 | 6.16 | 20.90 | 18.58 |
| TaMCA | 106.39 | 88.88 | 37.26 | 22.78 | 73.50 | 46.93 | 178.42 | 90.29 |
| TbMCA | 39.97 | 60.85 | 1411.00 | 3409.17 | 99.67 | 105.38 | 283.87 | 158.97 |
| THCA | 0.70 | 0.27 | 1.22 | 1.28 | 1.10 | 0.46 | 0.95 | 0.38 |
| TCA | 77.26 | 61.10 | 1090.38 | 2518.67 | 169.45 | 194.98 | 457.02 | 272.80 |

*


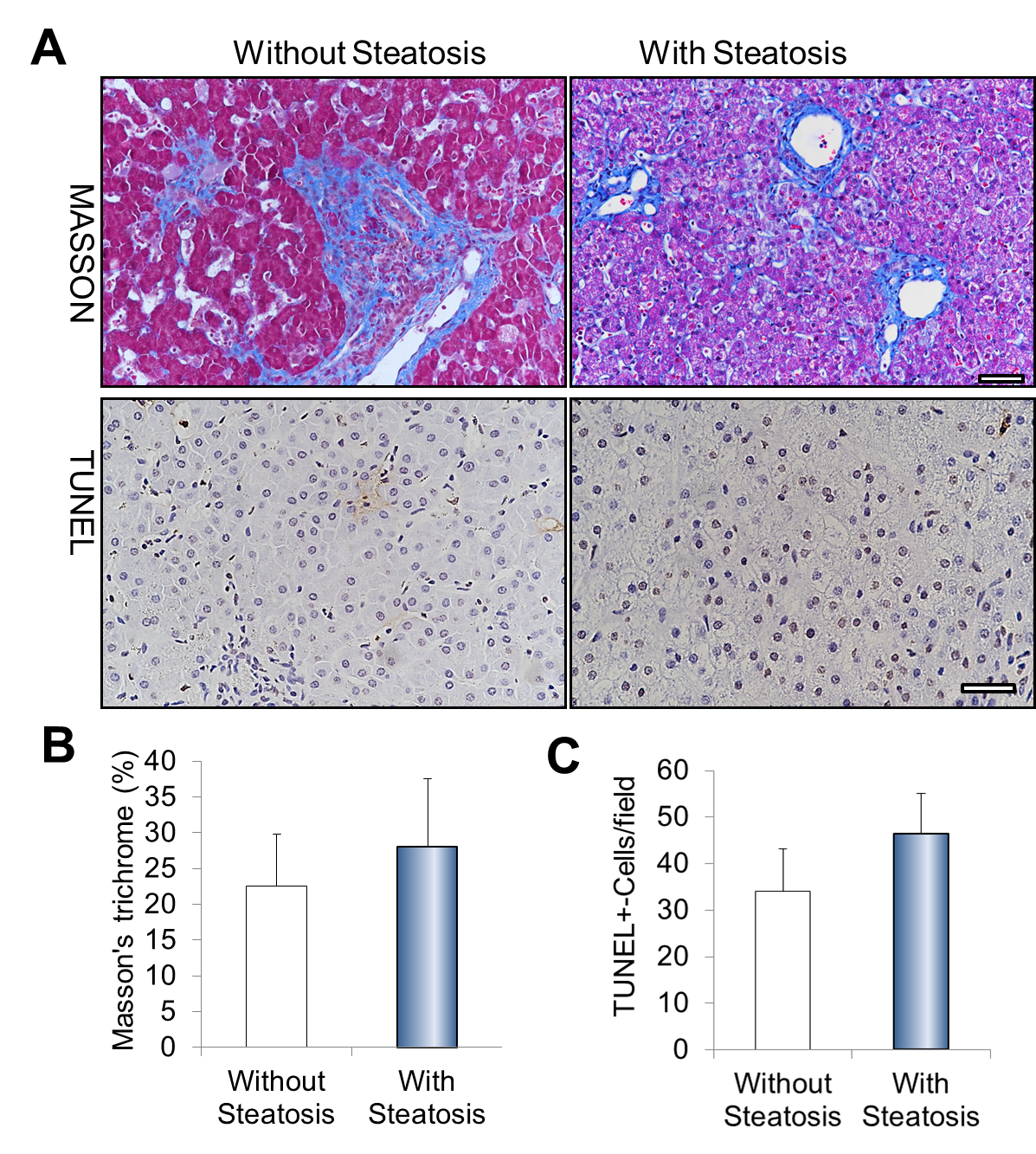


**Supplementary Figure 1. Liver histological alteration in pediatric IF patients** (A) Pediatric IF patients with liver steatosis have more hepatic apoptosis (TUNEL-stained, n=17) and fibrosis (Masson trichrome assay, n=24) than the ones whitout steatosis. (B) Quantification of Masson trichrome analysis. (C) Quantification of TUNEL-positive cells. cale bar = 50 μm. *, p <0.015;

**Supplementary Reference**

1. Li M, Wang W, Soroka CJ, Mennone A, Harry K, Weinman EJ*, et al.* NHERF-1 binds to Mrp2 and regulates hepatic Mrp2 expression and function. *The Journal of biological chemistry* 2010, **285**(25)**:** 19299-19307.

2. Bedossa P, Poynard T. An algorithm for the grading of activity in chronic hepatitis C. The METAVIR Cooperative Study Group. *Hepatology* 1996, **24**(2)**:** 289-293.

3. Xing H, Jia K, He J, Shi C, Fang M, Song L*, et al.* Establishment of the tree shrew as an alcohol-induced Fatty liver model for the study of alcoholic liver diseases. *PloS one* 2015, **10**(6)**:** e0128253.

4. Xie G, Zhong W, Li H, Li Q, Qiu Y, Zheng X*, et al.* Alteration of bile acid metabolism in the rat induced by chronic ethanol consumption. *FASEB J* 2013, **27**(9)**:** 3583-3593.

5. Garcia-Canaveras JC, Donato MT, Castell JV, Lahoz A. Targeted profiling of circulating and hepatic bile acids in human, mouse, and rat using a UPLC-MRM-MS-validated method. *J Lipid Res* 2012, **53**(10)**:** 2231-2241.
